# Immunization with live virus vaccine protects highly susceptible DBA/2J mice from lethal influenza A H1N1 infection

**DOI:** 10.1186/1743-422X-9-212

**Published:** 2012-09-19

**Authors:** Leonie Dengler, Mathias May, Esther Wilk, Mahmoud M Bahgat, Klaus Schughart

**Affiliations:** 1 Department of Infection Genetics, Helmholtz Centre for Infection Research and University of Veterinary Medicine Hannover, Inhoffenstr. 7, D-38124, Braunschweig, Germany

**Keywords:** Influenza A virus, Mouse, DBA/2J, Immunization

## Abstract

**Background:**

The mouse represents an important model system to study the host response to influenza A infections and to evaluate new prevention or treatment strategies. We and others reported that the susceptibility to influenza A virus infections strongly varies among different inbred mouse strains. In particular, DBA/2J mice are highly susceptible to several influenza A subtypes, including human isolates and exhibit severe symptoms after infection with clinical isolates.

**Findings:**

Upon intra-muscular immunization with live H1N1 influenza A virus (mouse-adapted PR8M, and 2009 pandemic human HA04), DBA/2J mice mounted virus-specific IgG responses and were protected against a subsequent lethal challenge. The immune response and rescue from death after immunization in DBA/2J was similar to those observed for C57BL/6J mice.

**Conclusions:**

DBA/2J mice represent a suitable mouse model to evaluate virulence and pathogenicity as well as immunization regimes against existing and newly emerging human influenza strains without the need for prior adaptation of the virus to the mouse.

## Findings

Influenza A virus infections are a serious health problem, not only during yearly epidemics but also for newly emerging pandemics [1-4]. The mouse has been shown to represent a valuable model system to evaluate the virulence and pathogenicity of presently circulating subtypes as well as newly emerging H5N1 and 2009 pandemic H1N1 subtypes (*e.g.*[5-14]). Bird viruses are able to infect the lungs of mice without prior adaptation but human isolates differ largely in their virulence in mice [15,16]. Studies in mice were initially performed in two inbred mouse strains, C57BL/6J and BALB/c. We and others demonstrated that the susceptibility to influenza virus infection largely varies among different inbred mouse strains [12,15,17-22]. In particular, DBA/2J mice are highly susceptible to infections with mouse-adapted viruses. But more importantly, they support viral replication and develop symptoms upon infection with several human and bird influenza isolates that were not adapted to the mouse species [15,16,23]. A total of 18 low-pathogenic non-mouse-adapted influenza isolates, including five human isolates, were tested in DBA/2J mice and more than 50% were pathogenic for DBA/2J whereas only two were pathogenic for C57BL/6J mice [15]. H3 and H4 subtypes were only low pathogenic whereas H5, H6, H7, H9, H10 subtypes were highly pathogenic in DBA/2J mice [15]. Infection of DBA/2J mice with different H1N1 avian isolates revealed that many were very virulent in DBA/2J but much less than in BALB/c mice, and that H2, H3, H4, H6, H10 and H12 subtypes were less pathogenic than H1N1 subtypes [16].

Thus, DBA/2J mice represent an ideal system to evaluate virulence and pathogenicity but also preventive and therapeutic interventions against existing and newly emerging human influenza strains. Here, we demonstrate that DBA/2J mice immunized intra-muscularly (i.m.) with live influenza H1N1 viruses developed an influenza-specific IgG response and were subsequently protected against lethal infections.

**Figure 1:**
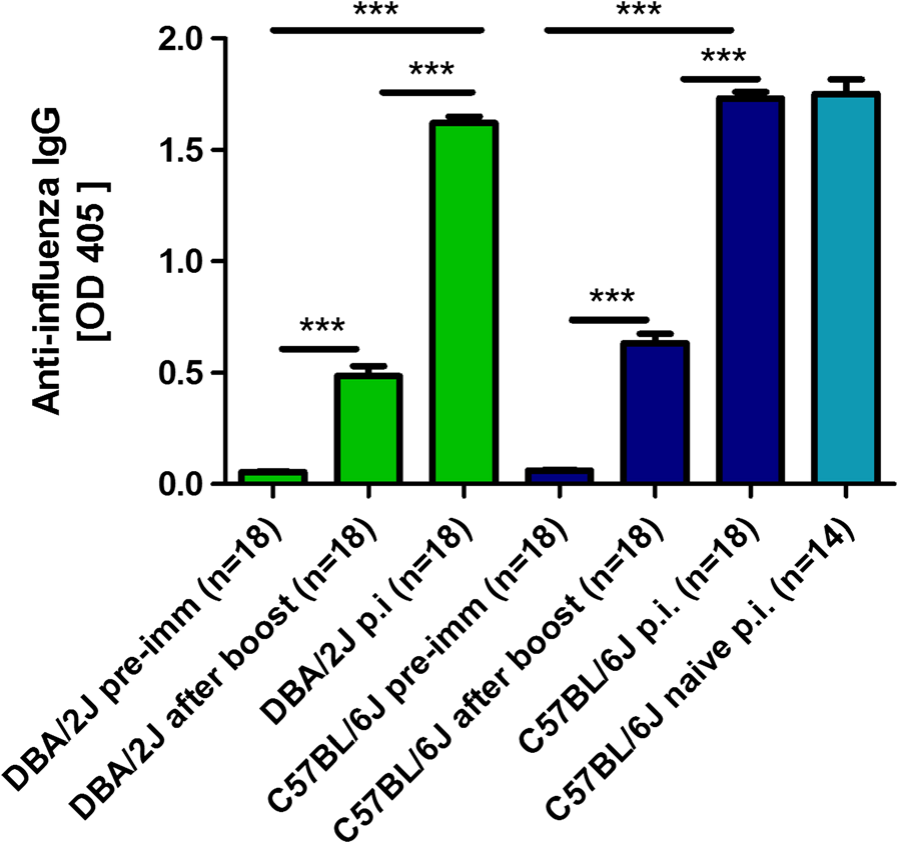
**DBA/2J and C57BL/6J mice mounted influenza-specific IgG titers after immunization with live PR8M virus.** Female DBA/2J and C57BL/6J mice were immunized by i.m. injection of 2 × 10^3^ FFU PR8M virus in 20 μl PBS. Injections were repeated after 14 days. Sera were taken before immunization (pre-imm) and 14 days after the booster immunization (after boost) by bleeding via the retro-orbital sinus. Mice were subsequently infected intra-nasally with 2 × 10^3^ FFU PR8M virus. Sera from surviving DBA/2J and C57BL/6J mice were collected by heart puncture 14 days after infection (p.i.). In addition, sera from non-immunized but infected C57BL/6J mice were collected by heart puncture 14 days after infection with 2 × 10^3^ FFU PR8M (naïve, p.i.). All sera were diluted 1:1000 and an ELISA for the presence of influenza-specific antibodies was performed. Absorbance at 405nm is shown. Responses in DBA/2J mice are indicated in green, responses in C57BL/6J mice in blue, respectively. Data represent mean values +/- SEM. The significance of differences between groups was tested using the Mann-Whitney-U-test, ***: p value < 0.0001.

Female DBA/2J and C57BL/6J mice were immunized at the age of 10-12 weeks by i.m. injection of 2 × 10^3^ focus forming units (FFU) [24] of mouse adapted A/PuertoRico/8/34 H1N1 virus (PR8M, Münster variant) in 20μl PBS, and a booster immunization 14 days later with the same dose of virus. It should be noted that different variants of the laboratory PR8 virus exist which differ in their virulence in mice [25]. Here, we used the PR8M (Münster) variant which is lethal for DBA/2J mice but not for C57BL/6J mice at an infection dose of 2 × 10^3^ FFU [17]. Fourteen days after the boost, mice were bled via the retro-orbital sinus. All sera were diluted 1:1000 and an ELISA was performed using plates that were coated with 1.6 × 10^5^ FFU PR8M virus/ml. For detection of virus specific IgG, peroxidase-labeled anti-mouse IgG (KPL; Gaithersburg, Madison, USA) was used as a secondary antibody and visualization of the reaction was carried out using a peroxidase specific substrate. As depicted in Figure 1, both DBA/2J and C57BL/6J mice exhibited significant levels of influenza-specific IgG levels after immunization compared to naive mice.

**Figure 2:**
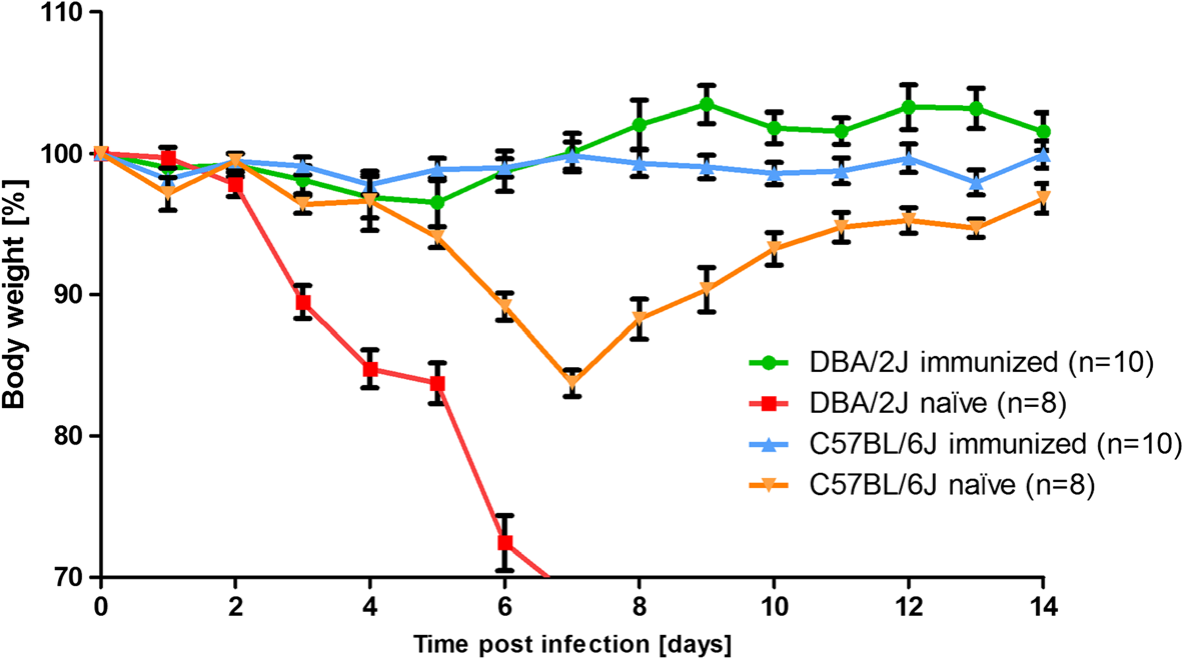
**Immunized DBA/2J mice did not lose body weight and survived lethal infection with mouse-adapted H1N1 influenza A virus.** Immunized and naïve female DBA/2J and C57BL/6J mice were infected with 2 *×* 10^3^ FFU/mouse PR8M virus. Body weight loss for each group of infected mice at various days p.i. (time post infection) is shown with reference to the starting weight (body weight [%]). In addition to mice that were found dead, mice with a weight loss of more than 30% of the starting body weight were euthanized and recorded as dead. Data represent mean values +/- SEM. Using the Mann-Whitney-U-test, body weight loss between immunized and naïve DBA/2J mice was significantly different (p value < 0.05) at day 3 to 6 p.i. Similarly, body weight loss between immunized and naïve C57BL/6J was significantly different at day 5 to 12 p.i. (p value < 0.05).

Two weeks after the boost, immunized and non-immunized DBA/2J mice were challenged by intra-nasal application with 2 × 10^3^ FFU PR8M virus representing a 55-fold lethal dose [17]. For the infection, mice were anesthetized by intra-peritoneal injection of a solution (10μl/g body weight) containing 85% NaCl (0.9%), 10% Ketamine (100 mg/ml), 5% Xylazine (20 mg/ml). Figure 2 illustrates that body weight loss of immunized DBA/2J mice was significantly different from non-immunized DBA/2J mice after infection. Indeed, immunized DBA/2J mice exhibited only very minor body weight loss after infection. Whereas all non-immunized DBA/2J mice succumbed to the infection between day 6 and 7 post infection (p.i.), all immunized mice survived. Furthermore, C57BL/6J mice were immunized with two i.m. injections of 2 × 10^3^ FFU PR8M virus, two weeks apart, and subsequently infected with 2 × 10^3^ FFU PR8M virus two weeks after the boost. The infection dose of the challenge is not lethal for C57BL/6J mice but causes significant body weight loss [17]. Similarly to DBA/2J mice, immunized C57BL/6J also exhibited significantly less reduced body weight loss compared to non-immunized C57BL/6J mice after infection (Figure 2).

A further increase in the influenza-specific antibody response was observed in immunized and infected DBA/2J and C57BL/6J compared to the titers measured after the booster immunization (Figure 1). The antibody titers in the immunized and infected C57BL/6J mice were comparable to non-immunized C57BL/6J mice that survived the infection (Figure 1).

**Figure 3:**
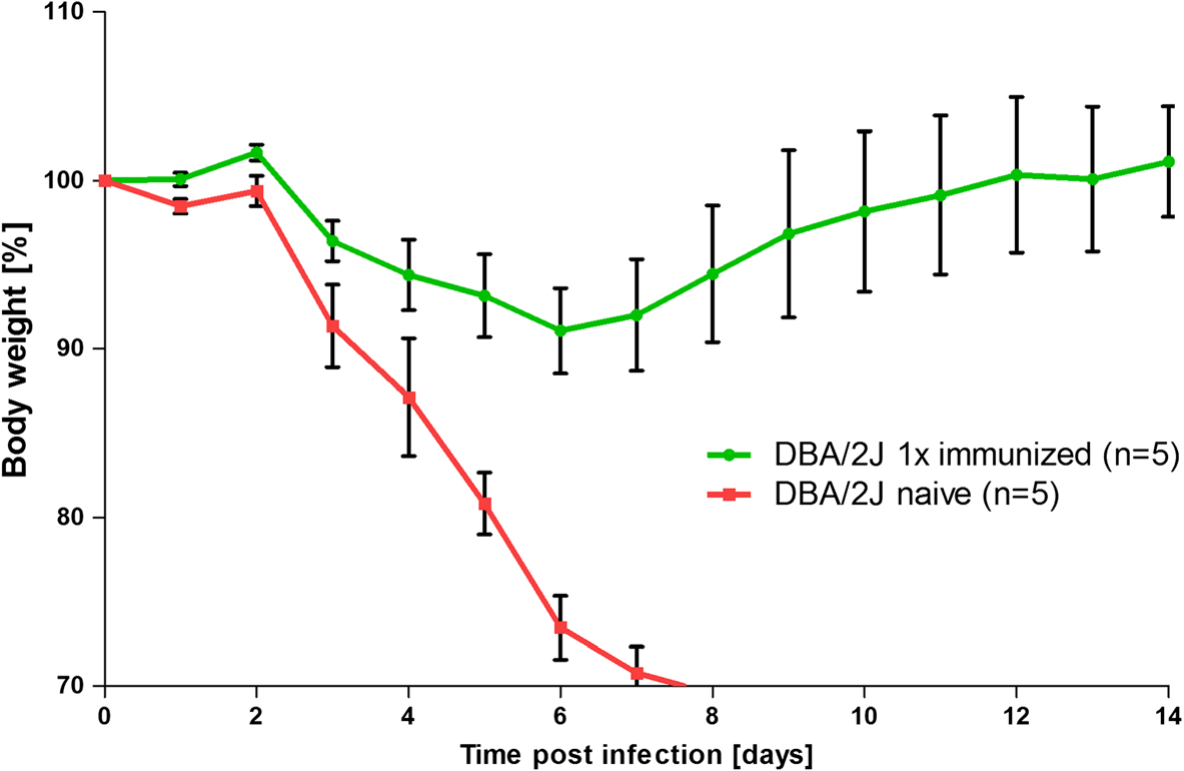
**A single i.m. immunization protects DBA/2J mice from subsequent lethal challenge.** Female DBA/2J mice were immunized once by i.m. injection of 2 *×* 10^3^ FFU PR8M virus (1*×* i.m.). Immunized and naïve DBA/2J mice were subsequently infected with 2 *×* 10^3^ FFU PR8M virus. Body weight loss for each group of mice at various days p.i. (time post infection) is shown with reference to the starting weight (body weight [%]). Data represent mean values +/- SEM. Using the Mann-Whitney-U-test, body weight loss between immunized and naïve DBA/2J mice was significantly different at day 5 to 7 p.i. (p < 0.05).

A single i.m. immunization of DBA/2J mice with 2 × 10^3^ FFU PR8M virus also resulted in an increase of the influenza-specific IgG response two weeks later (data not shown). Although the influenza-specific IgG levels were lower compared to two immunizations, these mice were fully protected from a lethal challenge, two weeks after immunization, with 2 × 10^3^ FFU PR8M virus (Figure 3).

**Figure 4:**
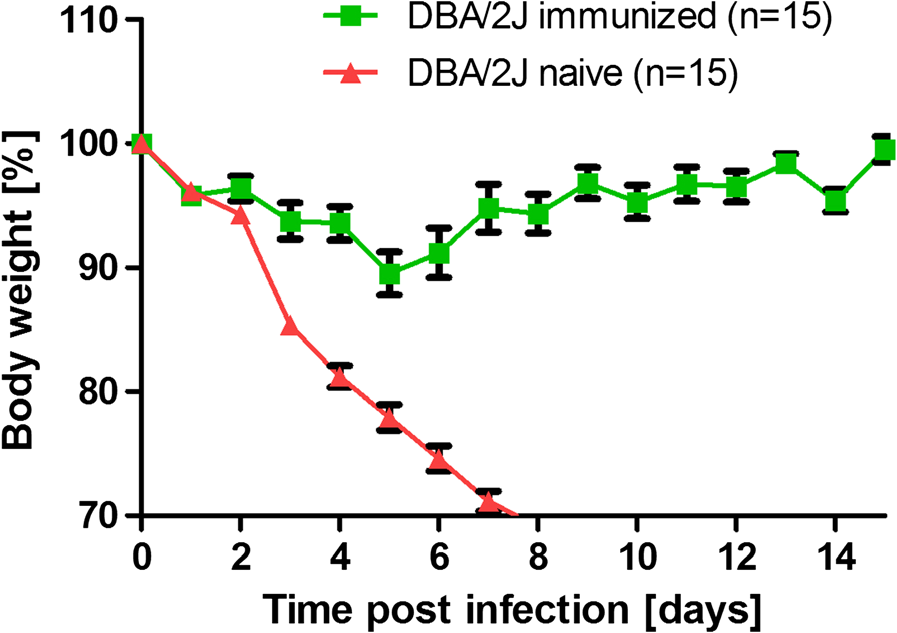
**Immunized DBA/2J mice did not lose body weight and survived lethal infection with human 2009 pandemic influenza A virus.** Female DBA/2J mice were immunized by i.m. injection of 2 *×* 10^5^ FFU HA04 virus in 20 μl PBS. Injections were repeated after 14 days. Immunized and naïve female DBA/2J mice were infected with 2 *×* 10^3^ FFU HA04 virus. Body weight loss for each group of infected mice at various days p.i. (time post infection) is shown with reference to the starting weight (body weight [%]). In addition to mice that were found dead, mice with a weight loss of more than 30% of the starting body weight were euthanized and recorded as dead. Data represent mean values +/- SEM.

In addition, we immunized DBA/2J mice by two i.m. injections (boosting 14 days after the first injection) with 2 × 10^5^ FFU of a human isolate of the pandemic swine influenza virus A/Hamburg/04/2009 (H1N1, HA04). Two weeks after the booster immunization, mice were challenged by intra-nasal application of 2 × 10^3^ FFU HA04 virus. Non-immunized mice rapidly lost body weight and died whereas all immunized mice exhibited a markedly reduced body weight loss and all infected mice survived (Figure 4).

Here, we demonstrated the proof-of principle for protective i.m. vaccination in DBA/2J mice using live influenza viruses which is very easy to perform because it does not require addition of adjuvants. These results, together with results from other groups [26,27] demonstrate that DBA/2J represents a very sensitive yet fully immuno-competent model system which is well suited to investigate adaptive host immune responses to influenza A virus from bird and human origin without the need for prior species-adaptation.

However, it should be noted that mouse knock-out lines are generally created on a C57BL/6N background [28] and, therefore, the function of a gene in a DBA/2J knock-out mutant line can only be tested after generating a congenic line by backcrossing.

Three other studies investigated the host response in DBA/2J mice after immunization and challenge with influenza A virus. Boon et al. showed that sera from humans containing cross-reactive antibodies against pandemic H1N1 virus protected DBA/2J mice from an infection with pandemic H1N1 [15]. Sambhara et al. immunized DBA/2J mice by subcutaneous injections with immunostimmulatory complexes containing influenza virus antigens and demonstrated that young and aged mice are better protected than control groups which were immunized with a split vaccine that is used in humans [27]. Solórzano et al., infected the lungs of DBA/2J mice with live-attenuated influenza virus and demonstrated that they are protected from lethal infection with pandemic human H1N1 virus [26].

In conclusion, our studies demonstrate that DBA/2J mice are capable of mounting a protective immune response against mouse-adapted as well as human isolates of H1N1 influenza virus. Together with previous studies, these results endorse the potential of DBA/2J mice as a highly valuable animal model system to evaluate vaccine strains and vaccination protocols against human influenza A virus strains without the need for species-adaptation. They extend previous studies by demonstrating that also i.m. injections of live virus are protective and thereby provide a simple method to evaluate cross-reactivity of vaccine strains.

## Ethics statement

All experiments in mice were approved by an external committee according to the national guidelines of the animal welfare law in Germany (‘Tierschutzgesetz in der Fassung der Bekanntmachung vom 18. Mai 2006 (BGBl. I S. 1206, 1313), das zuletzt durch Artikel 20 des Gesetzes vom 9. Dezember 2010 (BGBl. I S. 1934) geändert worden ist.’). The protocol used in these experiments has been reviewed by an ethics committee and approved by the ‘Niedersächsiches Landesamt für Verbraucherschutz und Lebensmittelsicherheit, Oldenburg, Germany’ (Permit Number: 33.9.42502-04-051/09).

## Competing interests

The authors declare that they have no competing interests.

## Authors’ contributions

LD and MM conducted the study, analyzed the results, and contributed to writing of the manuscript. KS and EW designed the study and wrote the manuscript. MMB established ELISA assays and contributed to the manuscript writing. All authors read and approved the final manuscript.
